# Intraoperative hemodynamics monitoring in a patient with pheochromocytoma multisystem crisis: a case report

**DOI:** 10.1186/s40981-018-0173-2

**Published:** 2018-05-01

**Authors:** Atsuko Kato, Hisayoshi Tamai, Kanji Uchida, Takamitsu Ikeda, Yoshitsugu Yamada

**Affiliations:** 1 Department of Anesthesiology, Graduate School of Medicine, The University of Tokyo, 7-3-1 Hongo, Bunkyo-ku, Tokyo, 113-8655 Japan; 2 Department of Anesthesiology, Toranomon Hospital, Tokyo, Japan

**Keywords:** Pheochromocytoma multisystem crisis, Pulmonary artery catheter-based cardiac output, Arterial pressure-based cardiac output, Stroke volume variation

## Abstract

**Background:**

Pheochromocytoma is a rare catecholamine-secreting tumor. To evaluate the intraoperative hemodynamics with precision is difficult.

**Case presentation:**

A 42-year-old man, who suddenly developed a life-threatening pheochromocytoma multisystem crisis that occurred during preoperative prophylactic medication, underwent urgent bilateral adrenalectomy. For the purpose of evaluating the intraoperative hemodynamics, we monitored both pulmonary artery catheter-based cardiac output (PACO) and arterial pressure-based cardiac output (APCO; FloTrac™). APCO fluctuated in poor agreement with the change in PACO, especially in the state of cytokine storming.

**Conclusions:**

Overall, the value of stroke volume variation derived from FloTrac™ changed in tandem with the intraoperative volume status, indicating its utility as a marker of circulatory hemodynamics.

## Background

Pheochromocytoma is a rare catecholamine-secreting tumor that originates from the adrenal medulla. Surgical resection of the tumor is the definitive treatment, but inadequate preoperative optimization of alpha- and/or beta-blockade can increase perioperative mortality and morbidity in patients with pheochromocytoma [1, 2]. The mortality can reach as high as 85% in the presence of a hyperadrenergic crisis [3–5], a life-threatening endocrine emergency leading to multiple organ failure, which is characterized by hypertensive crises, subsequent cardiogenic shock, pulmonary edema, acute liver and renal insufficiency, encephalopathy, and hyperthermia [2, 6]. Because of its severity, patients developing a hyperadrenergic crisis require preoperative prophylactic stabilization of circulatory hemodynamics [1, 2, 4, 5].

With marked elevation in the serum concentration of noradrenaline, accurate estimation of blood pressure and cardiac output is essential for successful anesthetic management. The use of a pulmonary artery catheter, which directly monitors cardiac output, contributes to the accurate estimation of the cardiovascular status [7, 8]. Alternatively, a less-invasive method for monitoring cardiac output based on the waveform of arterial blood pressure (ABP) has been introduced for clinical use [8]. In patients undergoing orthotopic liver transplantation, who could develop hyperdynamic circulation and low systemic vascular resistance, the arterial pressure-based cardiac output (APCO) had poor agreement with the pulmonary artery catheter-based cardiac output (PACO) during general anesthesia [9]. However, it remains unclear whether APCO is reliable in patients exhibiting elevated serum concentration of noradrenaline accompanied with increased vascular resistance [10].

Here, we present a case of a 42-year-old man who developed a hyperadrenergic crisis arising from pheochromocytoma and underwent emergent bilateral adrenalectomy. As part of intraoperative monitoring, we chose to employ two modalities with different algorithms, PACO and APCO, the former of which served as a de-facto standard. Our prediction was that APCO would detect the fluctuation of CO more promptly than PACO, thereby making it possible to respond to rapid changes in intraoperative hemodynamics. Written informed consent to publish was obtained from the patient.

## Case presentation

**Fig. 1:**
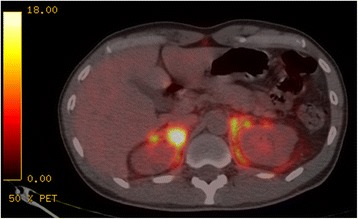
The iodine-123-metaiodobenzylguanidine (MIBG) scintigraphy image. MIBG scintigraphy showed increased uptake of MIBG in both adrenal glands, which was consistent with the diagnosis of bilateral pheochromocytomas

A 42-year-old man (175 cm in height, 61.8 kg in weight), with no medical and family history, experienced a temporal headache and nausea. He began to experience excessive sweating after meals and subsequently developed high blood pressure with the systolic pressure reaching 180 mmHg. Further examination revealed an elevated level of urinary noradrenaline excretion of 6460 μg/day (approximately 40 times above the upper limit of normal) and an increased serum concentration of interleukin-6 (IL-6) (74 ng/L, normally < 4.0 ng/L). Computed tomography (CT) images showed a 4.6-cm round mass on his right adrenal gland. Increased uptake of iodine-123-metaiodobenzylguanidine (MIBG) in both adrenal glands observed during iodine-123-MIBG scintigraphy and positron emission tomography (Fig. 1) led to the diagnosis of bilateral pheochromocytomas.

**Fig. 2:**
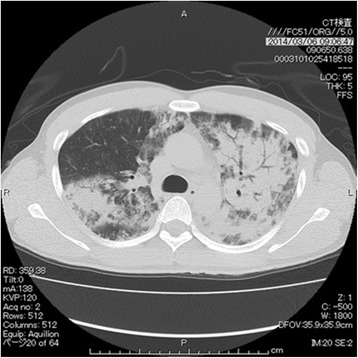
Computed tomography (CT) scan of the lung. CT images showed bilateral infiltration and air bronchograms

The patient was scheduled for surgical resection and was treated with a combination of bunazosin hydrochloride, phentolamine mesylate, and carvedilol to achieve prophylactic cardiovascular stabilization. Approximately 3 weeks later, he suddenly experienced dyspnea with a persistent fever peaking at 38.9 °C, and systemic administration of broad spectrum antibiotics was immediately initiated for a suspected bacterial pneumonia. However, the antibiotics were ineffective, and his respiratory condition deteriorated with PaO_2_ at 69 mmHg while receiving 5 L/min of oxygen via a face mask. Chest radiographs and CT images showed bilateral infiltration and air bronchograms, indicating pulmonary edema (Fig. 2). We found increased serum concentration of liver transaminases, suggesting hepatic insufficiency. Transthoracic echocardiography showed no signs of cardiac failure. The patient was suspected of developing pheochromocytoma multisystem crisis, a life-threatening condition that could cause permanent organ damage in a short span of time, and we thus decided to perform an emergency bilateral total adrenalectomy.

On arrival in the operating room, the vital signs were as follows: ABP 144/78 mmHg, heart rate 109 beats/min (sinus rhythm), SpO_2_ 95% (under 5 L/min of oxygen via a face mask), and body temperature 38.7 °C. General anesthesia was induced with target-controlled infusion (target-controlled infusion pump: TE-371 Telfusion ® TERUMO Co.Tokyo, Japan) of an effect site concentration of 2.5 μg/mL propofol, 200 μg of fentanyl, and 40 mg of rocuronium bromide to facilitate endotracheal intubation. Epidural anesthesia was waived because of his respiratory discomfort. General anesthesia was maintained with continuous infusion of propofol (constantly set at 2.6 μg/mL during the surgery with the bispectral index 45–63) and remifentanil (constantly set at 0.25 μg/kg/min) and intermittent supplementation of rocuronium bromide. The PaO_2_/FiO_2_ ratio was 72 at the beginning of the surgery.

The radial artery catheter was connected to the FloTrac™ sensor (third generation: version 3.06, Edwards Life Science, Irvine, CA, USA) to measure APCO along with arterial waveform and stroke volume variation (SVV). The pulmonary artery catheter, which was placed in the right jugular vein, was connected to the Vigilance™ monitor (Edwards Life Science, Irvine, CA, USA) to measure PACO using the thermodilution method. Phentolamine mesylate, landiolol hydrochloride, and prostaglandin E1 were continuously infused to stabilize blood pressure and heart rate. When the surgeons started to operate the right adrenal gland, ABP surged to 218/113 mmHg, which required an immediate bolus injection of phentolamine mesylate. Once the right adrenal gland vessels were ligated, ABP dropped to 70/30 mmHg and SVV jumped from 8 to 9% to 17%, probably as a result of systemic vasodilation and hypovolemia. Administration of a continuous infusion of norepinephrine (0.05–0.2 μg/kg/min) in combination with a rapid infusion of 1000 mL of albumin solution stabilized hemodynamics with ABP raising to 91/50 mmHg and SVV returning to less than 10%.

**Fig. 3:**
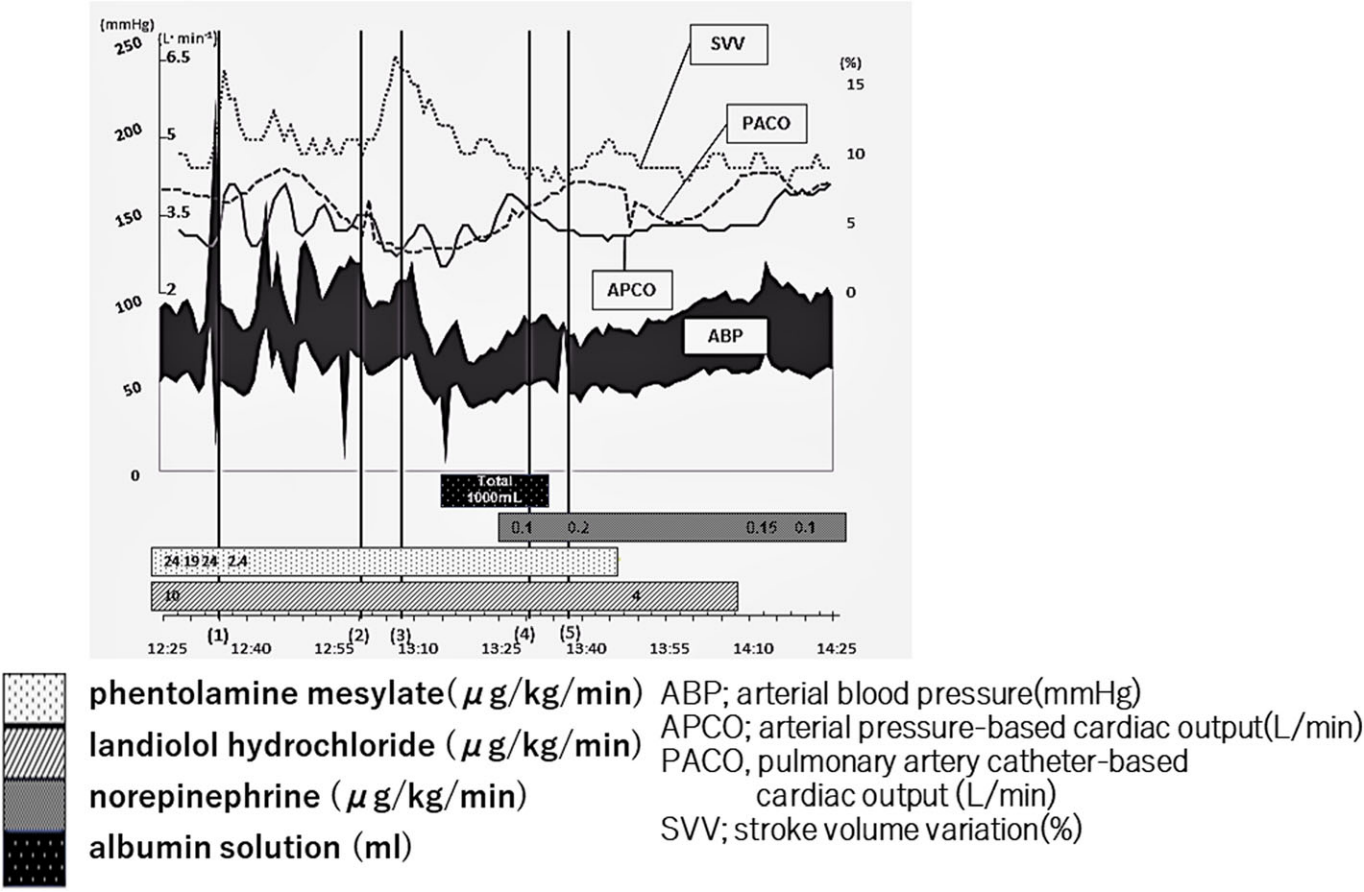
Chart showing the change in intraoperative hemodynamics with the values of circulatory monitors. Compared with pulmonary arterial catheter-based cardiac output (PACO), arterial waveform-based cardiac output (APCO) fluctuated more wildly in a different way from arterial blood pressure (ABP), especially while the right adrenal gland vessels were being resected, though the continuous infusion dose of propofol and remifentanil remained unchanged. (1) 12:35—there was a sudden surge in ABP immediately after the surgeons began operating the right adrenal gland, and a subsequent bolus injection of phentolamine mesylate decreased ABP. (2) 13:00—devascularization of the tumor was completed. (3) 13:08—stroke volume variation (SVV) increased from 10 to 17 after the devascularization. (4) 13:30—a rapid infusion of albumin stabilized circulatory hemodynamics. (5) 13:38—resection of the right adrenal gland tumor was completed

While the right adrenal gland was being operated, APCO fluctuated markedly in more or less inverse proportion to the change in ABP, whereas PACO shifted rather smoothly (Fig. 3). However, there was no appreciable difference between APCO and PACO while the left adrenal gland was being resected, the histological examination of which revealed no abnormal findings. After bilateral resection of the adrenal glands, ABP remained around 80/40 mmHg with a continuous infusion of norepinephrine (0.1 μg/kg/min). The total time required for surgery and general anesthesia were 214 and 308 min, respectively. The intraoperative fluid balance was + 1575 mL (5.1 mL/kg/h) with an estimated blood loss of 610 mL and urine output of 415 mL. The patient did not receive blood transfusions.

After surgery, he remained ventilated for 3 days in the intensive care unit (ICU). The PaO_2_/FiO_2_ ratio, which was initially 214, improved gradually over the next few days. Norepinephrine was tapered off, and the circulatory hemodynamics was stable thereafter. After being extubated on postoperative day (POD) 4, he was transferred to the ward on POD 5 and was uneventfully discharged on POD 14.

## Discussion

We presented a case of emergent bilateral adrenalectomy following pheochromocytoma multisystem crisis, for which we offered a successful anesthetic management using different types of circulatory monitors. In the present case, we used both PACO and APCO to evaluate cardiac output that could change drastically during the devascularization procedure mainly because of catecholamine release.

Before surgery, the patient developed non-cardiogenic pulmonary edema, which is rarely seen in those with pheochromocytoma [6]. Given that there was an abnormal increase in IL-6 levels, the pulmonary edema could have been attributed to the inflammatory mechanism, by which the overproduction of IL-6 caused from elevated catecholamine levels disrupts the endothelial junction, leading to capillary leakage [6]. Another pathophysiological mechanism includes the hemodynamic effect caused by increased alveolar capillary permeability resulting from post-capillary vasoconstriction of the lung.

The FloTrac™-derived value of APCO, which is calculated based on analysis of the peripheral arterial pressure waveform without external calibration, has theoretically a more rapid response than PACO. At the same time, however, APCO is vulnerable to changes in vascular resistance or artifacts, and there can be discordance between PACO and APCO, which have different algorithms [8, 10]. PACO requires more than 6 min to display the cardiac output on the monitor, and the subsequent value updates the average value every 7 min for every minute. In contrast, APCO (vol. 3.06) requires 40 s to measure cardiac output, and thereafter, it updates the average value every minute for every 20 s. We predicted that, theoretically, the use of APCO would lead to more accurate evaluation of intraoperative hemodynamics, but it turned out that APCO changed rather in poor agreement with PACO. Closer analysis of our case revealed that, compared with PACO, APCO apparently fluctuated almost in accordance with the change in ABP, with an approximate 5-min delay between 12:30 and 12:55. Nevertheless, considering that blood pressure is physiologically related to the product of cardiac output and total peripheral resistance, the trend in which APCO fluctuated inversely with ABP while the right adrenal gland was being operated might also be interpreted to reflect the degree of alfa-adrenergic vasoconstriction induced by the norepinephrine released from the tumor. This observation might suggest that APCO has the potential to be used as a cardiovascular parameter even in the presence of pheochromocytoma multisystem crisis, although the interpretation of APCO values should still be made with caution in patients with unstable hemodynamics.

In patients with pheochromocytoma, it is frequently difficult to accurately estimate the volume status. The value of SVV is nevertheless reliable even when the circulating plasma volume is diminished [11], as it is based on the magnitude of respiratory fluctuation of arterial pressure. In our case, SVV occasionally fluttered in the first half of the surgery, but its course was largely consistent with that of ABP when the right adrenal gland vessels were being operated. There was a sharp increase in SVV immediately after devascularization of the tumor, followed by a gradual decrease in accordance with the rapid infusion of albumin solution, but SVV was stable thereafter with a continuous infusion of norepinephrine. This observation could be related to the ability of SVV to reflect circulatory hemodynamics.

In conclusion, our case, pheochromocytoma multisystem crisis, adds evidence to the utility of SVV in determining the amount of fluid infusion even in conditions of hemodynamic instability. During the surgery, APCO was not closely associated with PACO especially when the serum concentration of noradrenaline was elevated.

## Abbreviations

ABP: Arterial blood pressure
APCO: Arterial pressure-based cardiac output
CT: Computed tomography
ICU: Intensive care unit
IL-6: Interleukin-6
MIBG: Metaiodobenzylguanidine
PACO: Pulmonary artery catheter-based cardiac output
POD: Postoperative day
SVV: Stroke volume variation
